# An Integrative Pharmacology Model for Decoding the Underlying Therapeutic Mechanisms of Ermiao Powder for Rheumatoid Arthritis

**DOI:** 10.3389/fphar.2022.801350

**Published:** 2022-02-23

**Authors:** Jie Wu, Kexin Wang, Qinwen Liu, Yi Li, Yingying Huang, Yujie Liu, Jieqi Cai, Chuanhui Yin, Xiaowei Li, Hailang Yu, Wei Meng, Handuo Wang, Aiping Lu, Yazi Li, Daogang Guan

**Affiliations:** ^1^ Department of Biochemistry and Molecular Biology, School of Basic Medical Sciences, Southern Medical University, Guangzhou, China; ^2^ Guangdong Provincial Key Laboratory of Single Cell Technology and Application, Guangzhou, China; ^3^ Neurosurgery Institute, Department of Neurosurgery, Zhujiang Hospital, Southern Medical University, Guangzhou, China; ^4^ Department of Radiology, Nanfang Hospital, Southern Medical University, Guangzhou, China; ^5^ Department of Obstetrics and Gynecology, Nanfang Hospital, Southern Medical University, Guangzhou, China; ^6^ Institute of Integrated Bioinformedicine and Translational Science, Hong Kong Baptist University, Hong Kong, China; ^7^ Guangdong-Hong Kong-Macau Joint Lab on Chinese Medicine and Immune Disease Research, Guangzhou, China

**Keywords:** rheumatoid arthritis (RA), traditional Chinese medicine (TCM), Ermiao Powder (EMP), coordinated functional components group (CFCG), coordinated molecular mechanisms

## Abstract

As a systemic inflammatory arthritis disease, rheumatoid arthritis (RA) is complex and hereditary. Traditional Chinese medicine (TCM) has evident advantages in treating complex diseases, and a variety of TCM formulas have been reported that have effective treatment on RA. Clinical and pharmacological studies showed that Ermiao Powder, which consists of *Phellodendron amurense* Rupr. (PAR) and *Atractylodes lancea* (*Thunb.*) DC. (ALD), can be used in the treatment of RA. Currently, most studies focus on the anti-inflammatory mechanism of PAR and ALD and are less focused on their coordinated molecular mechanism. In this research, we established an integrative pharmacological strategy to explore the coordinated molecular mechanism of the two herbs of Ermiao Powder in treating RA. To explore the potential coordinated mechanism of PAR and ALD, we firstly developed a novel mathematical model to calculate the contribution score of 126 active components and 85 active components, which contributed 90% of the total contribution scores that were retained to construct the coordinated functional space. Then, the knapsack algorithm was applied to identify the core coordinated functional components from the 85 active components. Finally, we obtained the potential coordinated functional components group (CFCG) with 37 components, including wogonin, paeonol, ethyl caffeate, and magnoflorine. Also, functional enrichment analysis was performed on the targets of CFCG to explore the potential coordinated molecular mechanisms of PAR and ALD. The results indicated that the CFCG could treat RA by coordinated targeting to the genes involved in immunity and inflammation-related signal pathways, such as phosphatidylinositol 3‑kinase/protein kinase B signaling pathway, mitogen-activated protein kinase signaling pathway, tumor necrosis factor signaling pathway, and nuclear factor-kappa B signaling pathway. The docking and *in vitro* experiments were used to predict the affinity and validate the effect of CFCG and further confirm the reliability of our method. Our integrative pharmacological strategy, including CFCG identification and verification, can provide the methodological references for exploring the coordinated mechanism of TCM in treating complex diseases and contribute to improving our understanding of the coordinated mechanism.

## Introduction

Rheumatoid arthritis (RA) is autoimmune arthritis with the characteristics of systemic inflammation, persistent synovitis, and autoantibodies (Scott et al., 2010; Wasserman 2011; Smolen et al., 2017). It is related to the aberrant immune network, which consists of complex inflammatory signaling pathways underlying its pathogenesis (Scott et al., 2010; Xu et al., 2018). The factors that contribute to RA are multiple and bring challenges to the treatment of RA (MacGregor et al., 2000; Wasserman 2011). This hereditary disease is common in the elderly and women (Scott et al., 2010). Early diagnosis of RA plays a key role in effective treatment. Also, in the earlier treatment, disease-modifying antirheumatic drugs (DMARDs) are applied (Quinn et al., 2001; Choi et al., 2002; Donahue et al., 2008). DMARDs are usually combined to control the disease (Simon 2000; Garrood and Scott 2001; Kremer 2001; O'Dell 2001; Choy et al., 2005). When DMARDs are not suitable for the patients, biological agents can be used to relieve the symptoms, such as tumor necrosis factor (TNF) inhibitors, abatacept, rituximab, and tocilizumab (Kristensen et al., 2007; Alonso-Ruiz et al., 2008; Singh et al., 2009; Scott et al., 2010). However, the costs of biological agents are high for patients, and they may have the risk of infections (Bansback et al., 2009; Leombruno et al., 2009; Strangfeld et al., 2009).

Traditional Chinese medicine (TCM) has a long history of development, and it has accumulated a large number of theories and rich clinical experience. TCM can effectively treat RA with high safety and few adverse effects (Shi et al., 2020). Increasing studies about RA have paid attention to the effects of TCM on the treatment of RA, and more and more evidence confirms that a variety of TCM formulas can have effective treatment on RA (Wang et al., 2011; Zhang et al., 2015; Guo et al., 2016; Cheng et al., 2017; Guo et al., 2017; Wang et al., 2018; Wang et al. 2020a; Wang et al. 2020b; Liu et al., 2020), such as Ermiao Powder (EMP) (Li et al., 2020), Danggui-Sini-decoction (Cheng et al., 2017; Wang L. et al., 2020), Huangqi-Guizhi Wuwu-Decoction (Wang et al., 2020a; Wang et al., 2020b; Liu et al., 2020), Bi-Qi capsule (Wang et al., 2011; Wang et al., 2018), Wu-Tou Decoction (Zhang et al., 2015; Guo et al., 2017), and Guizhi-Shaoyao-Zhimu (Guo et al., 2016). Among these formulas, EMP is widely used in clinical.

EMP is comprised of two herbs: *Phellodendron amurense* Rupr. (PAR) (15 g) and *Atractylodes lancea* (*Thunb.*) DC. (ALD) (15 g). Due to the function of anti-inflammatory, EMP has been widely used in treating RA. More studies focused on analyzing the anti-inflammatory mechanism of PAR and ALD separately or the anti-inflammatory effects of EMP. Chen et al. (2014) explored EMP's anti-inflammatory effects through the activation of NO and the production of pro-inflammatory cytokine in lipopolysaccharide (LPS)-induced RAW264.7 cells, and they found that EMP could inhibit the mitogen-activated protein kinase (MAPK) and nuclear factor-kappa B (NF-κB) pathway to decrease the inflammatory events and downregulate the expression of inducible nitric oxide synthase. To analyze EMP's effect on rats with RA, Li et al. (2020) tested joint swelling and arthritis index in different experimental groups of rats. The result of Masson staining showed that the joints of collagen-induced arthritis rats had pathological features of arthritis, such as synovial hyperplasia, articular cartilage, and bone erosion. The rats in the EMP group displayed significantly alleviated symptoms compared with the collagen-induced arthritis group, such as the swelling of the hind paws and the joint damage. Their results indicated that EMP could treat RA by regulating the cholinergic anti-inflammatory pathway (Li et al., 2020). However, the coordinated molecular mechanism of PAR and ALD in treating RA remains unclear.

Toward this end, we explored the coordinated molecular mechanisms of EMP in treating RA through a network pharmacology method. We screened active components from the components of PAR and ALD obtained from the Traditional Chinese Medicine Database and Analysis Platform (TCMSP), and 126 components that meet Lipinski's rules were retained. Then, the coordinated functional space prediction model was applied to quantify the effect of 126 active components on RA, and we identified the coordinated functional space composed of 85 components. Through the knapsack algorithm, we identified A coordinated functional components group (CFCG) from the 85 active components. We also explored the potential coordinated mechanism of CFCG by the functional enrichment analysis and molecular docking. The results indicated the potential co-functional effect of CFCG, and they can target genes related to inflammation and immunity. Finally, we validated the effect of CFCG through *in vitro* experiments. The results demonstrated the coordinated mechanism of CFCG from PAR and ALD in treating RA. Our study’s principal novelty is the design of a reliable optimization model and reverse-engineering strategy, which can obtain the core components group with the smallest number of components and the most extensive coverage of function from EMP. It could provide a methodologic reference for exploring the coordinated mechanism of TCM in treating complex diseases and prescriptions optimization.

## Methods

### Dataset Collection

The chemical components of PAR and ALD were collected from the TCMSP (https://tcmsp-e.com/) (Ru et al., 2014). Open Babel toolkit (version 2.4.1) was applied to convert the components' chemical structure to canonical SMILES (O'Boyle et al., 2011).

### Absorption, Distribution, Metabolism, and Excretion Screening

From SwissADME (http://www.swissadme.ch/index.php) (Daina et al., 2017), we obtained the properties of all components, including molecular weight (MW), rotatable bonds (RBN), number of hydrogen bonds acceptors (nHAcc), number of hydrogen bonds donors (nHDon), and the Consensus LogP (ClogP). Lipinski's rules were applied to filter active components of PAR and ALD: 1) MW lower than 500 Da, 2) RBN lower than 11, 3) nHAcc lower than 10, 4) nHDon lower than 5, and 5) the ClogP value over −2 and lower than 5 (Lipinski et al., 2001).

### Target Prediction

The target genes of active components were predicted through Similarity Ensemble Approach (SEA) (http://sea.bkslab.org/) (Keiser et al., 2007), HitPick (http://mips.helmholtz-muenchen.de/hitpick/cgi-bin/index.cgi?content=targetPrediction.html) (Liu et al., 2013), and SwissTargetPrediction (http://www.swisstargetprediction.ch/) (Gfeller et al., 2014). Then, the prediction results from the three databases were merged.

### Component-Target Networks Construction

The component-target (C-T) network was constructed based on active components and their target genes by Cytoscape (version 3.6.0) (Shannon et al., 2003). The Cytoscape plugin NetworkAnalyzer was then used to obtain and analyze the C-T networks’ topological properties.

### Coordinated Functional Space Prediction Model

To analyze the effect of each active component of PAR and ALD on RA, a coordinated functional space prediction model was constructed to calculate the contribution score of each component to the coordinated functional space as follows:

$$CSC\left(i\right)=\overset{n}{\underset{ij}{\sum }}\left(NL\left(C_{ij}\right)+NL\left(dose_{i}\right)\right)$$

$$NL\left(C_{ij}\right)=\frac{C_{ij}-C_{min}}{C_{max}-C_{min}}$$

$$NL\left(dose_{i}\right)=\frac{dose_{i}-dose_{min}}{dose_{max}-dose_{min}}$$

$$C_{ij}=Comp_{i}\times \left[\left(\frac{C_{edge}}{T_{edge}}+\left|\frac{Comp_{Hi}-Comp_{Ci}}{Comp_{Hi}+Comp_{Ci}}\right|\right)\times Tar_{j}\right]$$

In the equation discussed earlier, i represents the component, and j is one of its targets. For each component, CSC represents the contribution score, and Comp_i_ represents the eccentricity. Comp_Hi_ represents the eccentricity of each component only in PAR, and Comp_Ci_ represents components that only consist in ALD. C_edge_ is the edge count of each component, T_edge_ is the edge count of all the component’s targets, and Tar_j_ represents the sum eccentricity value of all the component’s targets. NL (C_ij_) represents the value of min–max normalization to C_ij_, and NL (dose_i_) represents the value of min–max normalization to each component’s dose in EMP.

According to the coordinated functional space prediction models, we obtained each component's contribution score. The top 85 components were selected; the sum of their contribution score accounts for 90% of the total contribution score of all components.

### Function Enrichment Analysis

Gene Ontology (GO) and Kyoto Encyclopedia of Genes and Genomes (KEGG) pathway analysis for targets of components in this study and RA-related genes was performed by R package “clusterProfiler,” respectively (Yu et al., 2012).

### Identification of Key Coordinated Function Components

To identify the core coordinated functional components of the top 85 active components that have the greatest effects in EMP, we applied the knapsack algorithm. For the 900 targets of the 85 active components, the target–target interaction was identified according to the protein–protein interaction (PPI) network. Then, the degrees of the 85 active components and the 900 targets were obtained by the Cytoscape plugin NetworkAnalyzer. The 473 targets whose degrees were over the median degree of 900 targets and 85 components were retained.

Next, the knapsack algorithm was performed for the 85 components and their 473 targets.

$$f_{iN}=\{\begin{array}{c} \;0\;\;\;\;\;\;\;\;\;\;\;\;\;\;\;\;\;\;\;\;\;\;\;\;\;\;\;\;\;\;\;\;\;\;\;\;\;\;\;\;\;\;\;\;\;\;\;\;\;\;\;\;\;\;\;\;\;\;\;\;\;\;\;\;\;\;\;\;\;\;\;\;\;\;\;\;\;\;\;\;\;\;\;\;\;\;i=0\;or\;N=0\;\;\;\;\;\;\;\\ \overset{i-1}{\underset{N<n_{i}}{\sum }}k_{i-1}D_{i-1}\;\;\;\;\;\;\;\;\;\;\;\;\;\;\;\;\;\;\;\;\;\;\;\;\;\;\;\;\;\;\;\;\;\;\;\;\;\;\;\;\;\;\;\;\;\;\;\;\;\;\;\;0<i<85\;\;\;\;\;\;\;\;\;\;\;\;\\ \text{max}\left\{f_{\left(i-1\right)N},\;f_{\left(i-1\right)\left(N-n_{i}\right)}+D_{i}\right\}\;0<i\leq 85,N\geq n_{i}\;\;\;\;\;\;\;\;\;\;\; \end{array}$$

$$k_{i}=0/1,i=1,2,\ldots ,85$$

In the formula, k_i_ represents the component i that was selected or not, and D_i_ is the sum degree of its targets. N is set to 426, which is the largest number of targets in the knapsack. n_i_ represents the count of targets of component i. f_iN_ represents the largest sum degrees of the targets of components in the knapsack, whereas the number of targets is N. In short, when the number of targets in the knapsack accounts for 90% (426) of the 473 targets, the number of components put in the knapsack is the least, and the sum of the degrees of these 426 targets is the largest. CFCG, which consists of 37 components, was identified.

### Prediction of Coordinated Mechanism by Molecular Docking

The three-dimensional conformer of CFCG was collected from ZINC (https://zinc.docking.org/) (Sterling and Irwin 2015) and PubChem (https://pubchem.ncbi.nlm.nih.gov) databases (Kim et al., 2021). The proteins coded by the target genes of CFCG were acquired from the protein data bank (http://www.rcsb.org) (wwPDB Consortium, 2019). Auto Dock Tools (Morris et al., 2009) and Autodock Vina (Trott and Olson 2010) were used to docking with the seed of docking set to 10,000, the energy range is four, and the exhaustiveness is 96. The affinity method and pyMOL (Delano, 2002; Mura et al., 2010) were used to estimate and perform the docking result.

## Experimental Verification of Coordinated Functional Components Group

### Materials

Wogonin (≥98% purity by high-performance liquid chromatography) was purchased from Jingzhu Biotechnology Co., Ltd. (Nanjing, China). Paeonol, magnoflorine (≥98% purity by high-performance liquid chromatography), and ethyl caffeate were purchased from Jiangsu Yongjian Pharmaceutical Co., Ltd. (Jiangsu, China). Moreover, fetal bovine serum and Dulbecco’s modified Eagle’s medium, which were required for the experiments, were obtained from Gibco (Grand Island, United States). Also, LPS was acquired from Sigma-Aldrich Co., Ltd. (St. Louis, United States).

### Cell Culture and Treatment

Mouse macrophage RAW264.7 cells were acquired from the cell bank of the Chinese Academy of Sciences (Shanghai, China). The RAW264.7 cells were cultured in complete Dulbecco’s modified Eagle’s medium with 8% fetal bovine serum, then incubated in a constant temperature incubator at 37°C with an atmosphere of 5% CO_2_. The culture medium needed to change every 1–2 days. RAW264.7 cells were blown down and passaged at a ratio of 1:2 or 1:3 when they had reached approximately 80% confluence. The cells (2 × 10^4^ per/well) were then seeded in 96-well plates until they reached 80% confluence. Next, the cells were treated with wogonin, paeonol, ethyl caffeate, and magnoflorine for 2 h and treated with LPS (1 μg/ml) for 24 h subsequently.

### Cell Viability Measure

The CCK-8 was used to assay the cell viability. To test the cytotoxicity of four components in our experiment, RAW264.7 cells were placed in 96-well plates with a density of 1 × 10^5^ cells/ml. The cells reached approximately 80% confluence after 24 h of incubation. Then, the cells were treated with various concentrations of wogonin (6.25, 12.5, 25, 50, and 100 μM), paeonol (10, 50, 100, 200, and 400 μM), ethyl caffeate (75, 150, 300, 500, and 800 nM), and magnoflorine (0.1, 1, 5, 10, and 50 μM). Subsequently, we added 10-μl CCK8 (Dojindo) in each well. After incubation for 3 h, cell viability (CCK8 activity) was quantified through the absorbance at 450 nm measured by a microplate reader (Tecan infinite M200).

### Assay the Content of NO

After 2 h of incubation of the RAW264.7 cells with caffeic acid, wogonin, paeonol, ethyl caffeate, and magnoflorine and 24 h of incubation with LPS (1 μg/ml), we collected the culture supernatant and mixed it with total nitric oxide assay kit (Beyotime) for NO assay. Then, the microplate reader (Tecan infinite M200) was used to assay the absorbance at 540 nm.

### Western Blot

RAW264.7cells were lysed in radioimmunoprecipitation assay lysis buffer (Beyotime, China) containing protease suppressor. The protein concentration was quantified by the bicinchoninic acid protein assay kit (Thermo Fisher Scientific, United States). An equal amount of protein was separated by sodium dodecyl sulfate–polyacrylamide gel electrophoresis and then transferred onto polyvinylidene fluoride membranes (Millipore, Bedford, MA). After blocking in 5% blocking in QuickBlock Western (Beyotime, cat. no. P0252) for 10 min, the proteins on the membrane were incubated with following primary antibodies at 4°C overnight: Akt (1:1,000; cat. no. 4691T; Cell Signaling Technology, United States), p-Akt (1:1,000; cat. no. 4060T; Cell Signaling Technology), p38 MAPK (1:1,000; cat. no. 8690T; Cell Signaling Technology), p-p38 MAPK (1:1,000; cat. no.4511T; Cell Signaling Technology), p44/42 MAPK (Erk1/2) (1:1,000; cat. no.4695T; Cell Signaling Technology), p-p44/42 MAPK (Erk1/2) (1:1,000; cat. no.4370T; Cell Signaling Technology), NF-κB p65 (1:1,000; cat. no. 8242T; Cell Signaling Technology), p-NF-κB p65 (1:1,000; cat. no. 3033T; Cell Signaling Technology), and TNF-α (1:1,000; cat. no. 17590-1-AP; Proteintech, United States). Following four washes in Tris-buffered saline with Tween, proteins were incubated with secondary antibodies for 1 h at room temperature. Antibody signal was detected using Clarity Western enhanced chemiluminescence substrate (Abbkine Scientific, China). The β-actin served as an endogenous reference.

### Statistical Analysis

To compare the anti-inflammatory effects of four components, GraphPad Prism 5 was used for statistical analysis. Student's t-test for the comparison of two groups was utilized to analyze the significance of differences, whereas a one-way analysis of variance followed by a Dunnett *post-hoc* test was used to compare more than two groups. Results were considered statistically significant if the *p*-value was <0.05.

## Results

**FIGURE 1:**
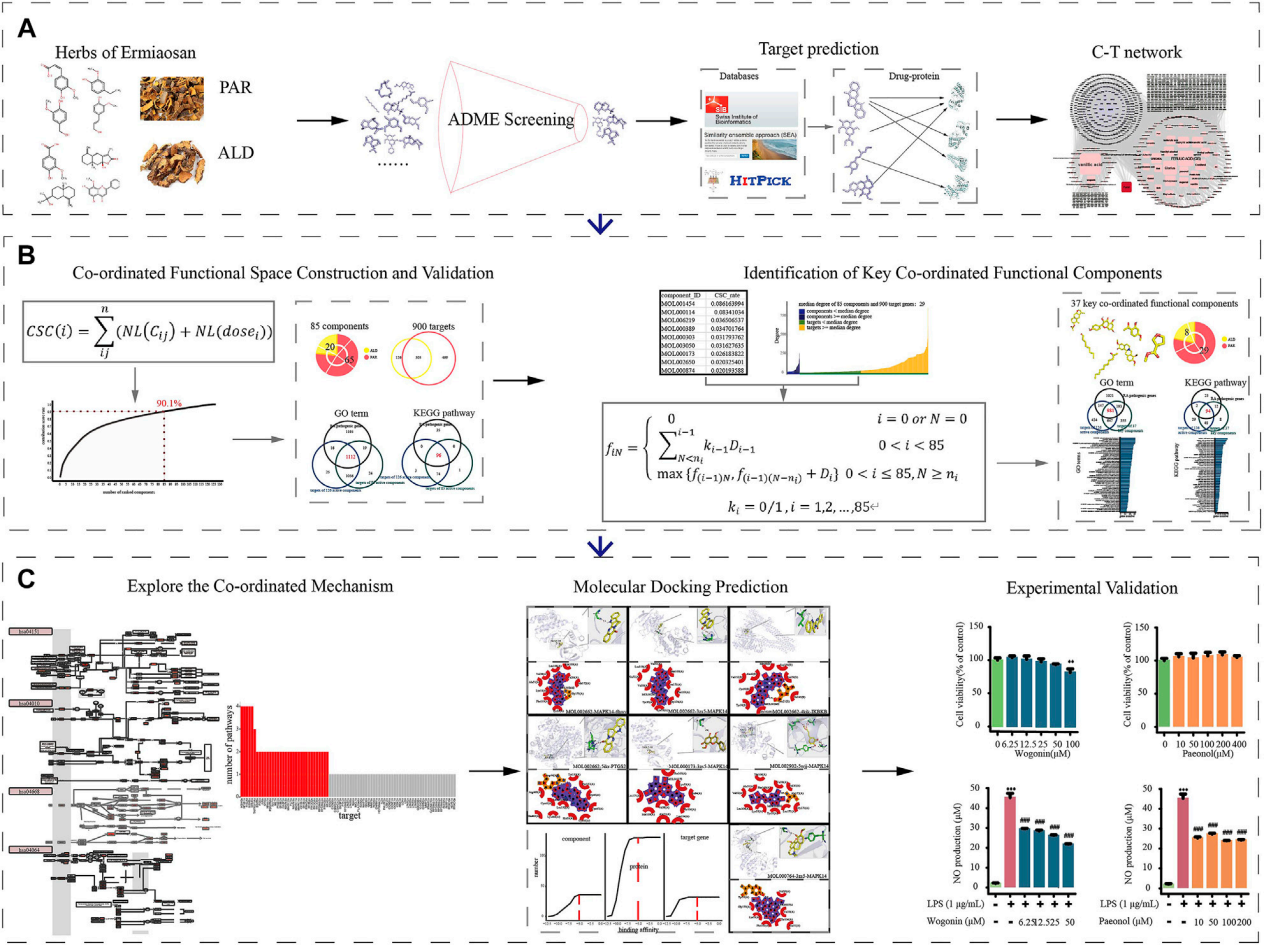
Workflow for systematic pharmacological strategy. Through integrative pharmacological strategy based on system pharmacological model, knapsack algorithm, molecular docking, and in vitro experiments, molecular mechanisms of ESM in treating RA were decoded and validated. **(A)** Components collection, ADME screening, target prediction and C-T networks construction. **(B)** Using the co-ordinated functional space prediction model and knapsack algorithm to identified the potential co-ordinated functional components group (CFCG). **(C)** Validation of CFCG and potential co-ordinated mechanism exploration.

An integrative pharmacological model explored the underlying therapeutic mechanism of PAR and ALD for RA. The components of the two herbs in EMP were obtained from the TCMSP. Next, the active components were screened by absorption, distribution, metabolism, and excretion. These active components’ targets were predicted by SEA, HitPick, and SwissTargetPrediction. The C-T networks were constructed by these components and their targets. Then, a coordinated functional space prediction model and knapsack algorithm were applied to demonstrate the co-functional mechanism of components from EMP in treating RA (Figure 1). Finally, we performed molecular docking to predict the co-functional effect of CFCG and further explore the coordinated mechanism. Also, the experiment results confirmed the reliability of CFCG, which was identified by our pharmacological strategy.

### Components in *Phellodendron amurense* Rupr. and *Atractylodes lancea* (*Thunb.*) DC.

For EMP, we collected 140 components of PAR and 49 of ALD from the TCSMP. Among the 187 components, 138 components were only in PAR, and 47 components were only in ALD, whereas two components were shared by the two herbs (Supplementary Table S1). It indicated the potential co-functional effect of the two herbs in EMP. The information of chemical components' identification and concentration of EMP was collected through searching the literature. As shown in Table 1, the information collected earlier provided an experiment-aided chemical space for the identification of active components in the following analysis.

**TABLE 1:** Information of EMP’s chemical components from literature.

Formula	Method	Component	Concentration	References
Ermiaosan	UPLC-MS/MS	Chlorogenic acid	3.884 ± 0.178 mg/g	Feng et al., 2017
		Ferulic acid	4.058 ± 0.09 mg/g	
		Berberine	69.857 ± 3.965 mg/g	
		Phellodendrine	7.002 ± 0.165 mg/g	
		Palmatine	1.045 ± 0.028 mg/g	
		Magnoflorine	2.221 ± 0.078 mg/g	
		Jatrorrhizine	0.894 ± 0.019 mg/g	
		Tetrahydropalmatine	0.0165 ± 0.0005 mg/g	
		Tetrahydroberberine	0.0035 ± 0.0005 mg/g	
		Obaculactone	3.244 ± 0.167 mg/g	
		Obacunone	0.213 ± 0.026 mg/g	
		Atractylenolide Ⅰ	0.3295 ± 0.0235 mg/g	
		Atractylenolide Ⅱ	0.131 ± 0.015 mg/g	
		AtractylenolideⅢ	0.144 ± 0.023 mg/g	

### Filtration of Active Components in *Phellodendron amurense* Rupr. and *Atractylodes lancea* (*Thunb.*) DC.

By integrating the components of PAR and ALD from the TCSMP and Table 1, 191 components were finally obtained. However, not all of the components have effective pharmacodynamic and pharmacokinetic characteristics in EMP. So, absorption, distribution, metabolism, and excretion screening was performed to filter out active components from them. As shown in Table 2, 100 active components of PAR and 27 of ALD were identified for the following analysis. Among the 126 active components, only Furol is shared by PAR and ALD. The result suggested that the components of the two herbs may play a co-functional effect on the treatment of RA *via* their specific space.

**TABLE 2:** The 126 active components ofactive components of PAR and ALD after ADME screening.

Molecule name	MW	RBN	nHAcc	nHDon	ClogP	Source
Undecenal	168.28	8	1	0	3.41	PAR
Furol	96.08	1	2	0	0.69	
Myrcene	136.23	4	0	0	3.43	
(S)-(+)-α-Phellandrene	136.23	1	0	0	2.97	
L-Limonen	136.23	1	0	0	3.35	
Eugenol	164.2	3	2	1	2.25	
Caprylic acid	144.21	6	2	1	2.23	
4-[(Z)-3-hydroxyprop-1-enyl]-2,6-dimethoxyphenol	210.23	4	4	2	1.47	
Ferulic Acid (CIS)	194.18	3	4	2	1.36	
Magnograndiolide	266.33	0	4	2	1.7	
Vanillin	152.15	2	3	1	1.2	
Pentylfuran	138.21	4	1	0	2.83	
WLN: VHR	106.12	1	1	0	1.57	
Trans-2-nonenal	140.22	6	1	0	2.66	
(2S,3S)-3,5,7-trihydroxy-2-(4-hydroxyphenyl)chroman-4-one	288.25	1	6	4	1	
Magnoflorine	342.41	2	4	2	1.89	
Menisporphine	321.33	3	5	0	2.98	
Palmatine	352.4	4	4	0	2.51	
STOCK1N-14407	355.43	4	5	0	3.1	
Fumarine	353.37	0	6	0	2.67	
Jatrorrizine	338.38	3	4	1	2.23	
Isocorypalmine	341.4	3	5	1	2.75	
Menisperine	356.44	3	4	1	2.19	
Paeonol	166.17	2	3	1	1.63	
Beta-elemene	204.35	3	0	0	4.65	
Mnk	170.29	8	1	0	3.48	
Phellamurin_qt	356.37	3	6	4	2.59	
Pisol	186.33	10	1	1	3.94	
Oxophorone	152.19	0	2	0	1.47	
Berberine	336.36	2	4	0	2.41	
(S)-Canadine	339.39	2	5	0	2.96	
Columbamine	338.38	3	4	1	2.23	
Coptisine	320.32	0	4	0	2.32	
EUG	150.17	2	2	1	2.14	
Isovanillin	152.15	2	3	1	1.12	
Methyl 3-furoate	126.11	2	3	0	1.06	
N-Methylflindersine	241.29	0	2	0	2.65	
Homocresol	152.19	2	2	1	2.02	
(s)-carvone	150.22	1	1	0	2.43	
Beta-Rhodinol	156.27	5	1	1	2.93	
Phlorol	122.16	1	1	1	2.11	
(±)-lyoniresinol	420.45	7	8	4	2	
Obacunoic acid	472.53	4	8	2	2.65	
Phellodendrine	342.41	2	4	2	1.68	
Phellopterin	300.31	4	5	0	3.36	
PEA	121.18	2	1	1	1.6	
Vanillyl alcohol	154.16	2	3	2	0.86	
(4R)-limonene 1beta, 2beta-epoxide	152.23	1	1	0	2.71	
Coniferol	180.2	3	3	2	1.62	
Dehydrotanshinone II A	292.33	0	3	0	3.61	
Delta7-Dehydrosophoramine	242.32	0	2	0	1.81	
Dictamine	199.21	1	3	0	2.63	
Kihadanin A	486.51	1	9	1	2	
Rutaecarpine	287.32	0	2	1	3.1	
Skimmianin	259.26	3	5	0	2.58	
Fagarine	229.23	2	4	0	2.59	
Ferulic Acid	192.21	3	3	2	2.15	
Chelerythrine	332.35	2	4	0	4.37	
Worenine	334.35	0	4	0	2.52	
Cavidine	353.41	2	5	0	3.23	
Hispidone	472.7	1	4	2	4.86	
Berberrubine	322.33	1	4	1	2.14	
Noroxyhydrastinine	191.18	0	3	1	1.23	
Ethyl caffeate	208.21	4	4	2	1.82	
Guasol	124.14	1	2	1	1.4	
IPH	94.11	0	1	1	1.41	
Nonanoic acid	158.24	7	2	1	2.6	
Dodec-2-enal	182.3	9	1	0	3.78	
Naphthalene	128.17	0	0	0	3.1	
Limonin	470.51	1	8	0	2.54	
5-Methylfurfural	110.11	1	2	0	1.02	
Maruzen M	122.16	1	1	1	2.11	
O-cresol	108.14	0	1	1	1.78	
Creosol	138.16	1	2	1	1.7	
Methyl naphthalene	142.2	0	0	0	3.46	
Isoferulic acid	194.18	3	4	2	1.39	
Cyclopentenone	82.1	0	1	0	0.96	
Methyl caffeate	194.18	3	4	2	1.35	
Clorius	136.15	2	2	0	1.84	
Ptelein	229.23	2	4	0	2.62	
SMR000232320	474.72	5	4	3	4.86	
Canthin-6-one	220.23	0	2	0	2.39	
4,10-dimethylene-7-isopropyl-5(E)-cyclodecenol	220.35	1	1	1	3.47	
4-[(1R,3aS,4R,6aS)-4-(4-hydroxy-3,5-dimethoxyphenyl)-1,3,3a,4,6,6a-hexahydrofuro (4,3-c)furan-1-yl]-2,6-dimethoxyphenol	418.44	6	8	2	2.33	
Guanidine	59.07	0	1	3	-1.01	
7-hydroxy-6-(2-hydroxyethyl)coumarin	206.19	2	4	2	1.35	
Thalifendine	322.33	1	4	1	2.14	
Furfuranol	98.1	1	2	1	0.62	
(S)-4-Nonanolide	156.22	4	2	0	2.24	
2,4,6-trimethyl-Octane	156.31	5	0	0	4.26	
Methyl atratate	196.2	2	4	2	1.77	
Acetylfuran	110.11	1	2	0	1.01	
Candicine	180.27	3	1	1	1.19	
2-undecenoic acid	184.28	8	2	1	3.18	
Homoveratrole	152.19	2	2	0	2.05	
Obacunone	454.51	1	7	0	3.19	
Auraptene	298.38	6	3	0	4.51	
Tetrahydropalmatine	355.43	4	5	0	3.08	
Jatrorrhizine	380.46	6	4	1	3.05	
Obaculactone	470.51	1	8	0	2.54	
Alpha-humulene	204.35	0	0	0	4.26	ALD
Beta-Eudesmol	222.37	1	1	1	3.61	
2-[(1R,3S,4S)-3-isopropenyl-4-methyl-4-vinylcyclohexyl]propan-2-ol	222.37	3	1	1	3.77	
Atractylenolide i	230.3	0	2	0	3.25	
Atractylenolide II	232.32	0	2	0	3.2	
Selina-4(14),7(11)-dien-8-one	218.33	0	1	0	3.65	
Vanillic acid	168.15	2	4	2	1.08	
Beta-Chamigrene	204.35	0	0	0	4.39	
Atractylone	216.32	0	1	0	3.81	
2-[(2S,5S,6S)-6,10-dimethylspiro [4.5]dec-9-en-2-yl]propan-2-ol	222.37	1	1	1	3.54	
ZINC01609418	222.37	4	1	1	3.76	
3β-hydroxyatractylone	232.32	0	2	1	2.87	
()-2-Carene	136.23	0	0	0	3.12	
Alpha-Guaiene	204.35	1	0	0	4.3	
Guaiene	204.35	0	0	0	4.23	
Guaiol	222.37	1	1	1	3.42	
Furol	96.08	1	2	0	0.69	
Wogonin	284.26	2	5	2	2.54	
Cyperene	204.35	0	0	0	4.4	
Atractylenolide iii	248.32	0	3	1	2.65	
2-Hydroxyisoxypropyl-3-hydroxy-7-isopentene-2,3-dihydrobenzofuran-5-carboxylic	306.35	4	5	3	2.37	
Beta-Eudesmol	222.37	1	1	1	3.61	
Butenolide B	234.29	0	3	1	2.21	
3β-acetoxyatractylone	274.35	2	3	0	3.3	
3,5-dimethoxy-4-glucosyloxyphenylallylalcohol_qt	210.23	4	4	2	1.55	
(Z)-caryophyllene	204.35	0	0	0	4.25	
Patchoulene	204.35	0	0	0	4.35	

### Component-Target Networks Construction

**FIGURE 2:**
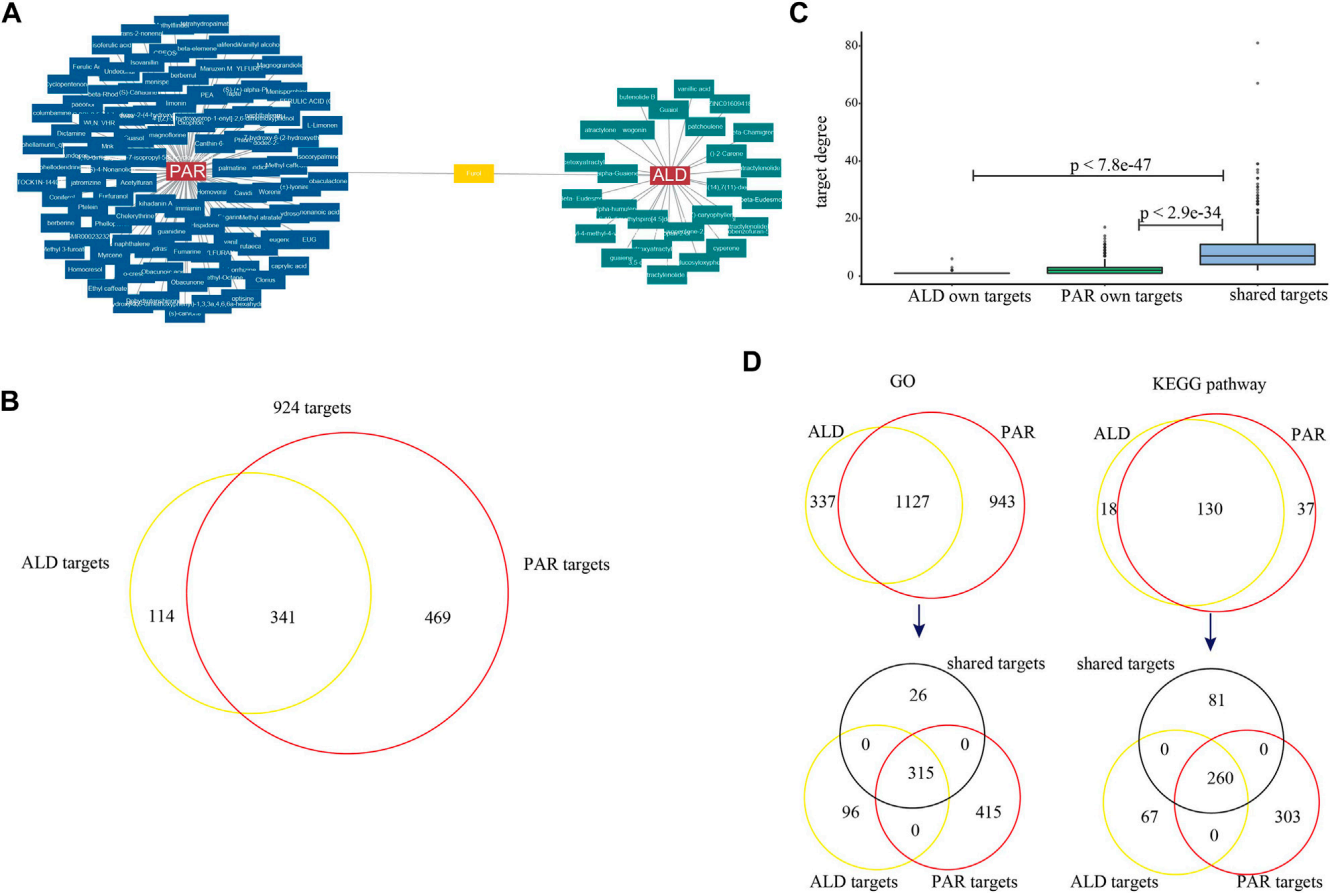
C-T networks of 127 active components and their target genes. **(A)** Active components of *Phellodendron amurense* Rupr. (PAR) and *Atractylodes lancea* (Thunb.) DC. (ALD). **(B)** Venn diagrams for target genes of active components of two herbs. **(C)** Box plots for degrees of targets in C-T network. **(D)** Venn diagrams for GO terms and KEGG pathways mapped by target genes of two herbs. Two Venn diagrams show target genes enriched in 1,127 GO terms and 130 KEGG pathways (black represents 341 shared target genes of two herbs, yellow represents genes targeted by ALD, and red represents genes targeted by PAR).

To explore whether the potential coordinated functional space of PAR and ALD exists, we constructed the C-T networks composed of active components and their targets (Figure 2). The targets of the 126 active components were obtained from SEA, HitPick, and SwissTargetPrediction. The PAR C-T network consisted of 3,839 components–targets, which included 100 active components and 810 targets. The ALD C-T network contains 776 components–targets, including 27 active components and 455 targets. The C-T networks indicated that one component might target multiple genes, and several components may intend to act on the same target. We also found that 341 targets were shared by the PAR C-T network and the ALD C-T network (Figures 2B,C), suggesting that some active components of PAR and some of ALD play roles on the same targets together. Also, in the C-T network constructed by 126 components and 924 targets, the degrees of these 341 targets were significantly greater than other targets (Student's t-test, 341 targets *vs*. PAR own targets, *p* < 2.9e^−34^; 341 targets *vs*. ALD own targets, *p* < 7.8e^−47^) (Figures 2B,C). We then performed GO and KEGG pathway enrichment analysis for the targets of the two herbs, respectively, and the results showed that 1,127 GO terms and 130 KEGG pathways were shared by the targets of the two herbs (Figure 2D). In the shared GO terms and pathways, we found that 92.4 and 76.2% of the 341 shared targets were enriched, respectively. These results revealed that PAR and ALD might play the principal co-functional effect through their shared target genes, which is consistent with the complex multi-target and multicomponent mediated coordinated mechanism of TCM.

### Construction and Validation of Coordinated Functional Space

**FIGURE 3:**
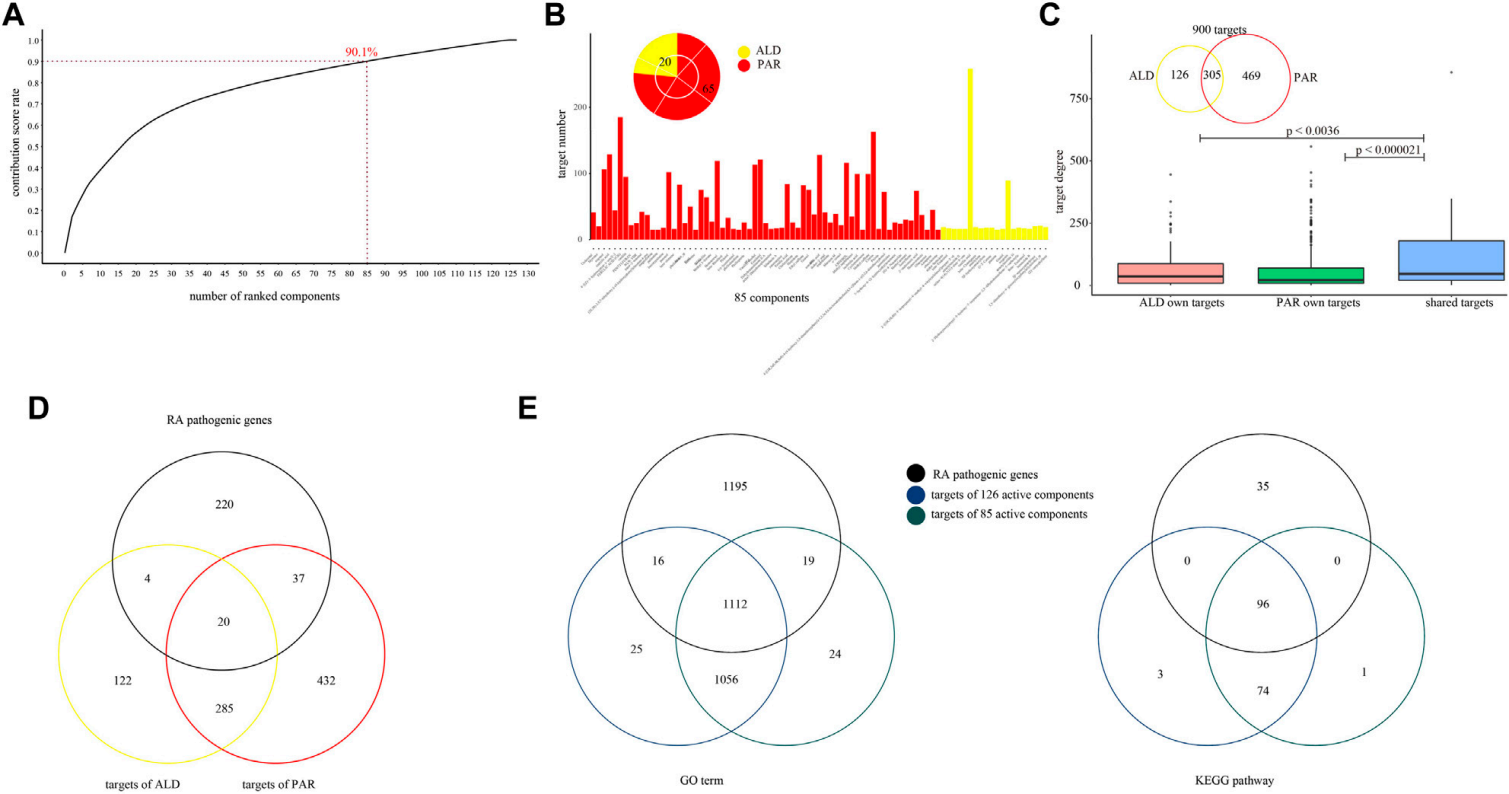
Coordinated functional space constructed by 85 active components. **(A)** Coordinated functional space prediction models obtained top 85 active components with 90.1% of sum contribution score. **(B)** Number of 85 active components’ targets. Among 85 components, 65 come from PAR, and 20 belong to ALD. **(C)** Degree of 85 active components’ targets in network converged by PPI network and C-T network. **(D)** Venn diagrams for two herbs’ targets and RA pathogenic genes. **(E)** Venn diagrams for GO-BP terms and KEGG pathways with RA pathogenic genes, 126 components' targets, and 85 components' targets mapped in.

To further analyze the co-functional effect of active components from PAR and ALD on RA, coordinated functional space prediction models were constructed to quantify the effect of each component. According to the results of models, the top 85 active components have the greatest effects of EMP on RA with 90.1% of the sum contribution score of 126 components (Figure 3A, Supplementary Table S1). Among the 14 components in Table 1, nine components (including berberine, phellodendrine, palmatine, magnoflorine, jatrorrhizine, ferulic acid, obaculactone, obacunone, and atractylenolideⅢ) were included in the 85 active components. We also found that the number of the 85 components' targets covers 97.4% of 126 components (900 of 924). These results indicated that the 85 components constitute the coordinated functional space of PAR and ALD in EMP. Among the 85 components, 65 come from PAR with 774 of 900 targets, and 20 belong to ALD with 431 of 900 targets, whereas 305 targets were shared by the components of the two herbs (Figures 3B,C). We further converged the PPI network and C-T network, which included 85 components and 900 targets, and the result showed that the degrees of the shared 305 targets were significantly greater than others (Student's t-test, 305 targets *vs*. PAR own targets, *p* < 0.000021; 341 targets *vs*. ALD own targets, *p* < 0.0036) (Figure 3C). It also demonstrated the co-functional effect of PAR and ALD in EMP on RA and indicated that the two herbs might perform their potential coordinated effect through their shared target genes.

To explore the potential effect of the 85 active components, we performed GO and KEGG pathway analysis for RA pathogenic genes, 924 targets of the 126 active components and 900 targets of the 85 active components, respectively. The RA pathogenic genes set contains 281 known pathogenic genes which have been confirmed by at least five previous studies. We found that the targets of 85 components contain 61 of the 281 RA pathogenic genes (Figure 3D). The results showed that 924 target genes of 126 active components were enriched in 2,209 GO-BP terms and mapped in 173 KEGG pathways and shared 1,128 GO-BP terms and 96 KEGG pathways with the RA pathogenic genes. Also, among these 1,128 GO-BP terms and 96 KEGG pathways, 98.6 and 100% of them were mapped by 900 targets of 85 active components, respectively (Figure 3E). These results suggested that the coordinated functional space consisted of 85 active components that have a potential correlation with RA pathogenic genes and may play an important role in EMP treatment of RA.

### Identification of Key Coordinated Functional Components From Coordinated Functional Space of Ermiao Powder

**FIGURE 4:**
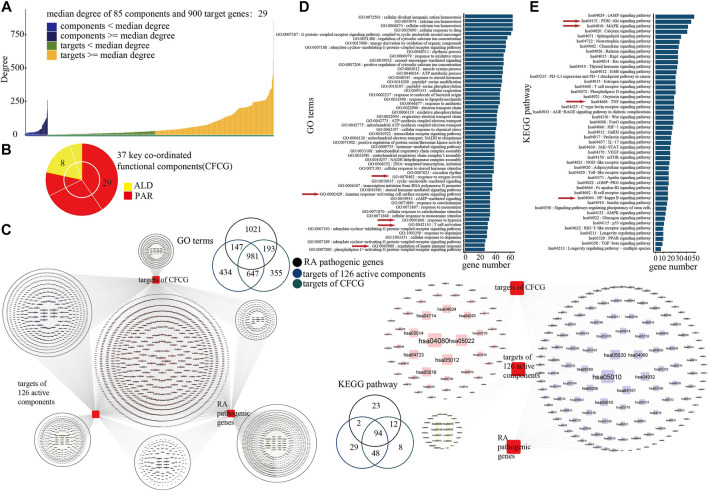
Identification of key coordinated functional components group (CFCG). **(A)** Degrees of 85 active components and their 900 target genes in network. Median degree of network was 29, and 473 targets with degrees over median degree were retained for following analysis. **(B)** CFCG composed of 37 active components. Among them, 29 components belong to PAR, and eight belong to ALD. **(C)** GO-BP terms and KEGG pathways with RA pathogenic genes, 126 components’ targets, and CFCG’s targets mapped in. **(D,E)** Results of GO **(D)** and KEGG pathway **(E)** enrichment analysis performed on target genes of CFCG.

It is known that the characteristics of the TCM mechanism are multicomponent and multi-target; the results of our analysis for the co-functional effect of PAR and ALD in EMP demonstrated the phenomenon. We found that 305 of the 85 actives components' targets were shared by components in PAR and others in ALD (Figure 3B). The result indicated that the 85 active components contain the main co-functional components, and further analysis was needed to identify the key co-functional components of EMP. According to the network converged by PPI network and C-T network, the 473 key targets with degrees over the median degree of the 900 targets and 85 components were retained for the following analysis (Figure 4A).

Based on the knapsack algorithm performed for the 85 components and their 473 key targets, we identified 37 active components as the key coordinated functional components (Figure 4B). GO and KEGG pathway analysis was performed for the 426 targets of the CFCG. The 426 targets were enriched in 2,176 GO-BP terms and mapped in 162 KEGG pathways, and they mapped in 86.96 and 97.92% of GO-BP terms and KEGG pathway of the overlap of RA-related genes and 924 targets of 126 active components (Figure 4C). The results demonstrated that the CFCG retained the main functions of the formulas of EMP and had the main coordinated effects in the treatment of EMP on RA.

### Analysis of Coordinated Mechanisms

To further explore the co-functional effects of CFCG, we dissect the results of GO and KEGG pathway enrichment analysis performed on the 426 targets (Figures 4D,E).

The results of the GO analysis showed that the targets were enriched in biological processes which play an important role in RA, such as response to oxygen levels (GO:0070482), response to hypoxia (GO:0001666), regulation of innate immune response (GO:0045088), immune response-activating cell surface receptor signaling pathway (GO:0002429), and T cell activation (GO:0042110) (Hu et al., 2014; Torices et al., 2017; Bartlett et al., 2018; Gong et al., 2019; Leite Pereira et al., 2019) (Figure 4D). RA is a systemic autoimmune inflammatory disease, which is related to the aberrant immune network (Xu et al., 2018). The inflammation of the synovium of the joint is the main pathological feature of RA. Angiogenesis plays a major role in the development of synovitis, whereas the hypoxic environment of the RA joint is closely related to the excessive formation of synovial vessels (Elshabrawy et al., 2015; Gong et al., 2019). CFCG may inhibit angiogenesis and reduce the production and expression of inflammatory cytokines by targeting the hypoxia-inducible factor-1α. Angiogenesis can contribute to the infiltration of inflammatory cells (such as neutrophils, monocytes, and macrophages) into the joint, and the pro-inflammatory environment can enhance the pathology of RA by extending the lifespan of inflammatory cells in the joint. Inhibition of joint angiogenesis can reduce the inflammation of the synovium. CFCG may also inhibit the inflammatory response and repair the damage of RA through regulating the expression of targets involved in immunity and inflammation, such as interleukin (IL)-6 and NF-κB. The results indicated that the CFCG could treat RA by targeting the genes related to inflammation and immunity.

The result of KEGG pathway enrichment analysis also indicated that the targets were involved in pathways related to inflammation and immunity, such as the PI3K-Akt signaling pathway (hsa04151), MAPK signaling pathway (hsa04010), TNF signaling pathway (hsa04668), NF-κB signaling pathway (hsa04064), T cell receptor (TCR) signaling pathway (hsa04660), and cyclic adenosine 3',5'-monophosphate signaling pathway (hsa04024) (Figure 4E).

**FIGURE 5:**
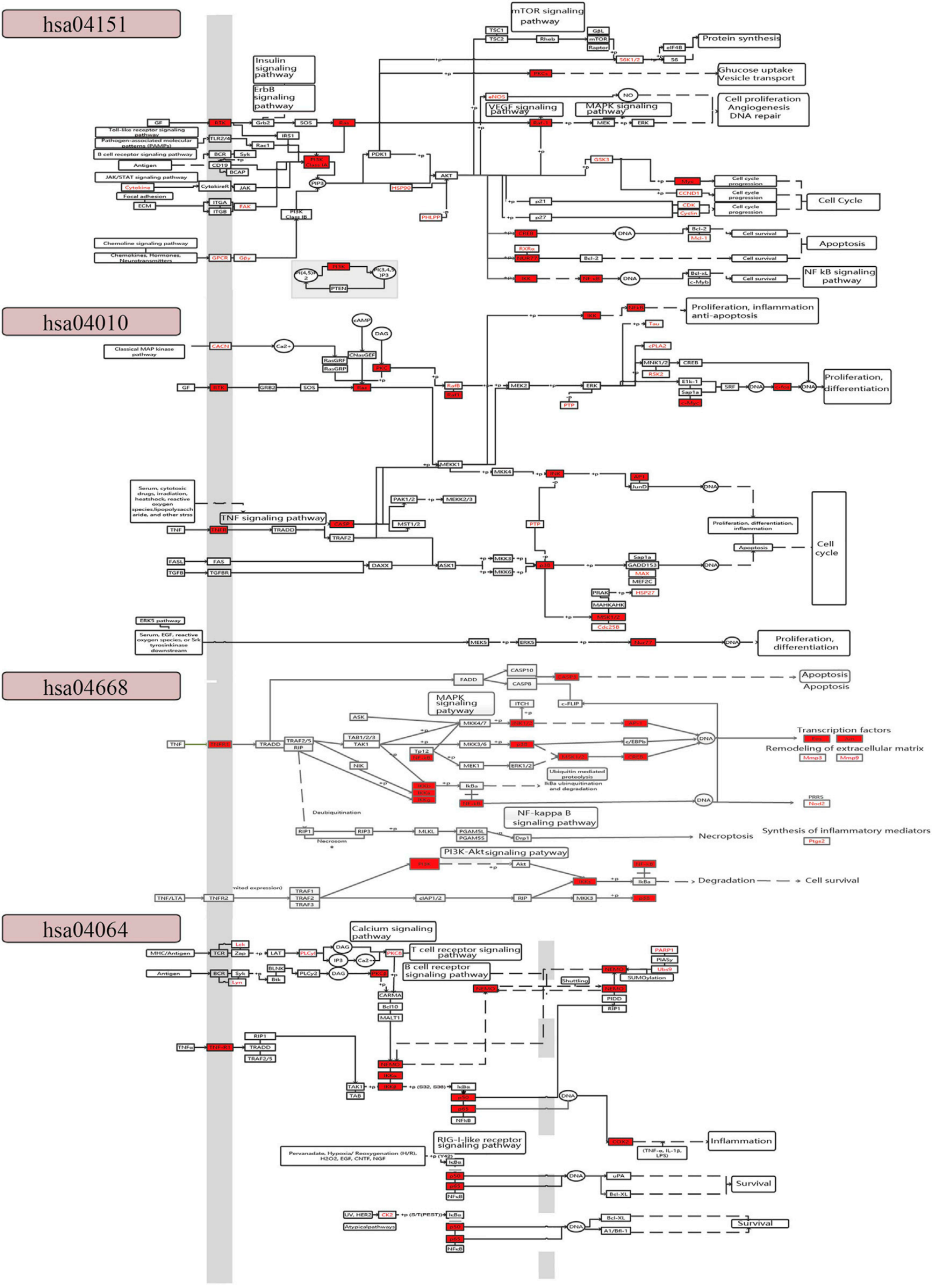
Comprehensive pathway with four pathways: hsa04151, hsa04010, hsa04668, and hsa04064.

To further explore the coordinated mechanism of the CFCG in treating RA, we constructed a comprehensive pathway with four pathways, including hsa04151, hsa04010, hsa04668, and hsa04064 (Figure 5). We defined the first three columns of targets as upstream target genes and others as downstream targets in the comprehensive pathway. The CFCG can regulate upstream targets of the PI3K-Akt signaling pathway such as RTK and FAK and downstream targets such as Ras, PI3K, Raf-1, Myc, CREB, IKK, and NF-κB. These targets are involved in cell proliferation, cell cycle, cell survival, and so on, which are involved in the development and progression of RA. The targets in the MARK signaling pathway are mainly associated with cell proliferation, differentiation, apoptosis, and inflammation, such as RTK, TNFR, Ras, CASP, p38, JNK, and Nur77. In the TNF signaling pathway, the CFCG can regulate targets such as TNFR1, NF-κB, p38, PI3K, Fos, Jun, and ptgs2 to affect cell survival and synthesis of inflammatory mediators. In the NF-κB signaling pathway, the proteins involved in immunity, inflammation, and cell survival are targeted by the CFCG, such as TNFR1, NEMO, p50, p65, and COX2. All the targets mentioned earlier play different roles in the four pathways, which indicate the coordinated therapeutic effect of the CFCG from PAR and ALD in treating RA.

### Prediction of Coordinated Mechanism by Molecular Docking

**FIGURE 6:**
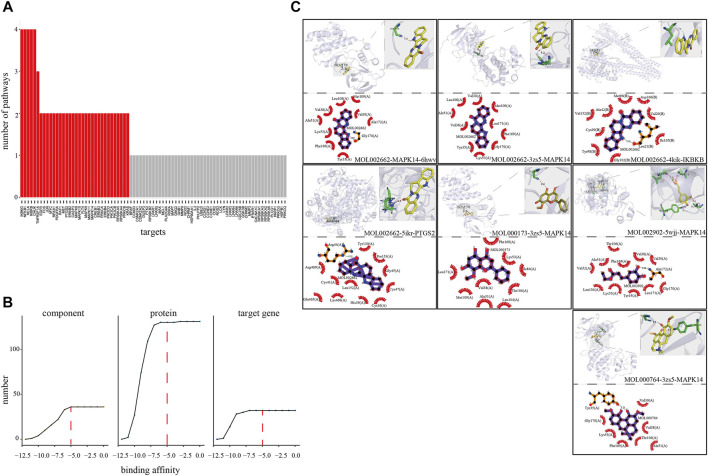
Verification of coordinated mechanism by molecular docking. **(A)** Number of pathways where target mapped in four pathways (hsa04151, hsa04010, hsa04668, and hsa04064). **(B)** Screening threshold of effective binding affinity. **(C)** Docking result visualization.

To explore the co-functional effect of the CFCG, we also conducted molecular docking for 37 components with a three-dimensional conformer and 131 proteins coded by the 32 target genes (Figures 6A–C). A total of 42,842 binding relationships in the docking results were obtained. Each binding relationship has a binding affinity value, and the smaller affinity value represents better binding. Among them, 25,802 binding relationships with binding affinity < −5 were retained, where components could bind with proteins effectively. The 25,802 binding relationships contained 37 components, 32 genes, and 130 proteins. Among these bindings, MOL002662 can bind best with protein 6 hwv coded by gene MAPK14 with the binding affinity of −11.2 kcal/mmol, followed by MOL002662-3zs5-MAPK14 (−11 kcal/mmol) and MOL002662-4kik-IKBKB (−10.8 kcal/mmol). Among the 37 components in 25,802 binding relationships, MOL000173 and MOL002662 could bind the most, with 130 proteins coded by 32 genes, whereas MOL000764 could bind the 127 proteins coded by 32 genes, MOL002902 could bind the 122 proteins coded by 31 genes, MOL000874 could bind the 106 proteins coded by 28 genes. MAPK14 and PTGS2 could bind the most, with 36 components. These results indicated that the CFCG could effectively bind with the proteins in the comprehensive pathway and suggested their potential co-functional effect in the treatment of RA.

### Verification of Coordinated Functional Components Group by *In Vitro* Experiment

To further validate the results of the identification of CFCG, four components (wogonin, paeonol, ethyl caffeate, and magnoflorine) from CFCG were selected for *in vitro* experiments. We detected the four components’ anti-inflammatory effects through RAW264.7 cells induced by LPS.

**FIGURE 7:**
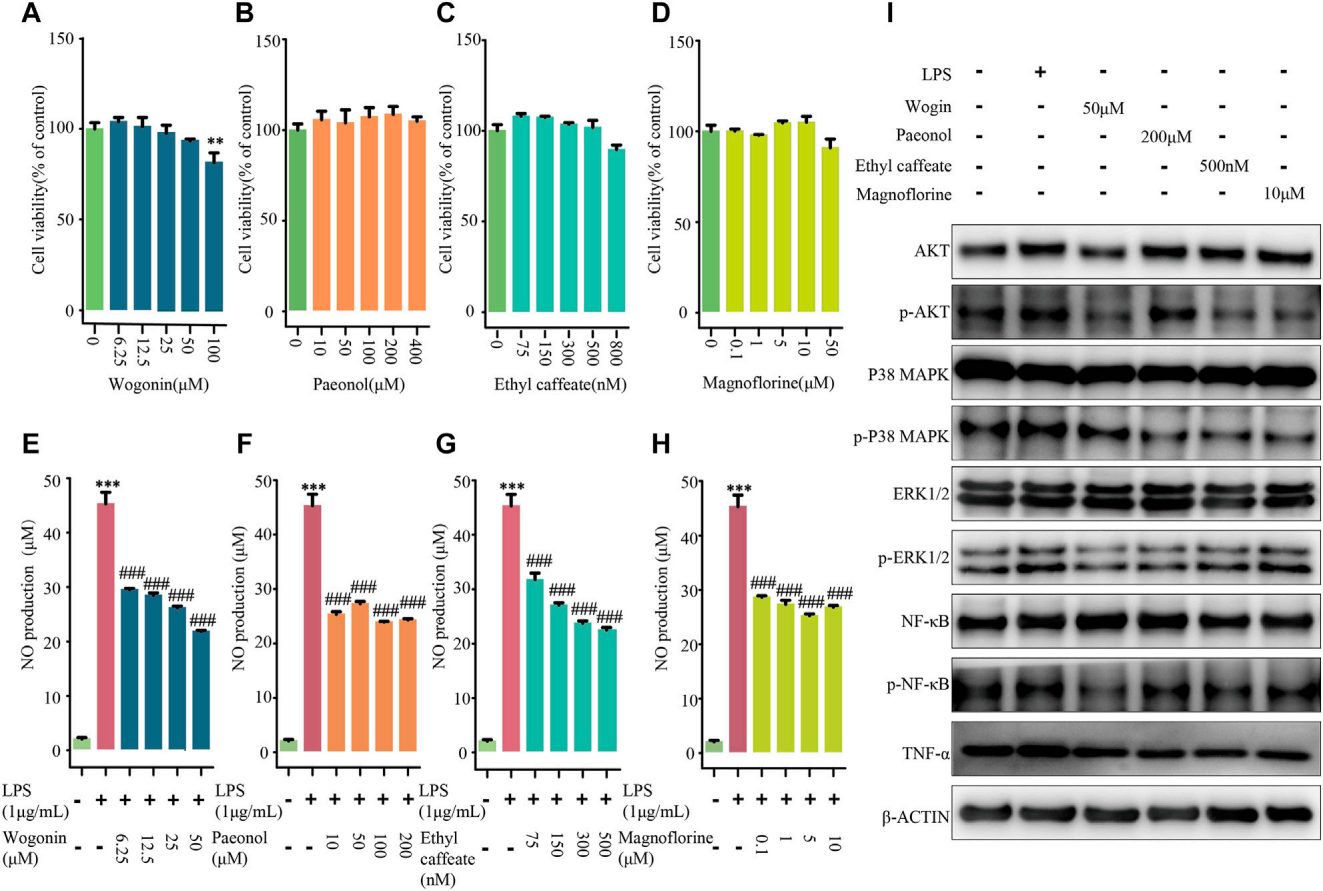
Experiments validation of four components from CFCG. Effects of wogonin **(A,E)**, paeonol **(B,F)**, ethyl caffeate **(C,G)**, and magnoflorine **(D,H)** on viabilities and NO production of RAW264.7 cells induced by LPS. **p < 0.01, ***p < 0.001 compared with control group. ^###^ *p* < 0.001 compared with LPS group. **(I)** LPS-induced RAW264.7 cells were treated with wogonin (50 μM), paeonol (200 μM), ethyl caffeate (500 nM), and magnoflorine (10 μM). Also, Western blotting was performed to detect activities of four pathways in RAW264.7 cells, including PI3K-Akt signaling pathway, MAPK signaling pathway, NF-κB signaling pathway, and TNF signaling pathway.

NO assay was applied to detect the effects of wogonin, paeonol, ethyl caffeate, and magnoflorine with different concentrations on RAW264.7 cells' viability. As compared with the control group, the four concentrations of wogonin (6.25, 12.5, 25, and 50 μM), paeonol (10, 50, 100, and 200 μM), ethyl caffeate (75, 150, 300, and 500 nM), and magnoflorine (0.1, 1, 5, and 10 μM) had no influence on cell viability; the four concentrations of these components were used for following experiments (Figures 7A–D).

Compared with the control group, we found that the NO level significantly increased by 2,034.2% in the culture medium of LPS-treated cells, whereas wogonin (6.25, 12.5, 25, and 50 μM), paeonol (10, 50, 100, and 200 μM), ethyl caffeate (75, 150, 300, and 500 nM), and magnoflorine (0.1, 1, 5, and 10 μM) significantly decreased the extracellular NO levels with *p* < 0.0001 in a concentration-dependent manner (Figures 7E–H). The results demonstrated that wogonin, paeonol, ethyl caffeate, and magnoflorine could effectively reduce the production of NO in RAW264.7 cells induced by LPS.

Furthermore, to validate the results of the comprehensive pathway, Western blotting was applied to detect the activities of the PI3K-Akt signaling pathway, MAPK signaling pathway, NF-κB signaling pathway, and TNF signaling pathway in RAW264.7 cells (Figure 7I). Compared with the control group, it was observed that the four pathways were all activated in LPS-induced RAW264.7 cells. However, the expression level of p-Akt, p-p38 MAPK, p-Erk1/2, p-NF-κB, and TNF-α were decreased in dosing groups, which supported that wogonin (50 μM), paeonol (200 μM), ethyl caffeate (500 nM), and magnoflorine (10 μM) can inhibit the activities of the four pathways in LPS-induced RAW264.7 cells. The results indicated that the CFCG plays a role in treating RA at least partially *via* reducing the activities of the PI3K-Akt signaling pathway, MAPK signaling pathway, TNF signaling pathway, and NF-κB signaling pathway. Also, it also validated the reliability and accuracy of the identification of CFCG.

## Discussion

As a chronic and systemic autoimmune inflammatory disease (Scott et al., 2010; Wasserman 2011; Smolen et al., 2017), the multiple factors contributing to RA and its complex pathogenesis bring challenges for therapeutic interventions. TCM has evident advantages in treating complex diseases, such as multi-target and multicomponent treatment (Wang L. et al., 2020; Shi et al., 2020; Yang et al., 2021). EMP is comprised of PAR and ALD, which have been widely applied in the treatment of rheumatic diseases (Resch et al., 1998; Kim et al., 2011; Xian et al., 2011; Tang et al., 2018; Wan 2018; Zhang et al., 2018; Li et al., 2020; Zhang et al., 2021). Clinical and pharmacological studies have demonstrated PAR's function as an anti-inflammatory (Kim et al., 2011; Xian et al., 2011; Wan 2018; Zhang et al., 2018). ALD can exhibit anti-inflammatory activity by inhibiting MPO activity and inflammatory cell infiltration and reducing the secretion of TNF-α, IL-1β, and IL-6 (Resch et al., 1998; Tang et al., 2018; Zhang et al., 2021). Currently, most studies have focused on analyzing the anti-inflammatory effects of PAR and ALD separately and the anti-inflammatory effects of EMP. Due to the unclear coordinated molecular mechanism of PAR and ALD in treating RA, we systematically explored the mechanisms of EMP in treating RA based on a network pharmacology method.

Recently, the flourishing development of network pharmacology has contributed to an emerging trend of TCM network pharmacology (Hao da and Xiao 2014; Zhou et al., 2020). TCM formula contains multiple herbs and treats complex diseases with the characteristic of multi-targets and multiomponents, which is consistent with the multi-target action network characteristic of network pharmacology. Many studies have been made in mechanism research of TCM by network pharmacology approach, while mostly inferred the potential mechanism by decoding the network constructed by the selected active components and their targets. For example, Tao et al. (2013) decoded the mechanism of the Curcumae Radix formula for preventing cardiovascular and cerebrovascular diseases based on 58 active components and 32 potential targets related to cerebrovascular diseases. Based on network pharmacology assays, Hong et al. (2017) constructed two drug-target networks of herb-induced liver injury of Xiao-Chai-Hu-Tang and Polygoni Multiflori Radix (Heshouwu) and identified the three potential hepatotoxic components from the networks. Zhou et al. (2018) applied network pharmacology and molecular docking technology to identify possible active components in Qingdai and explored their molecular mechanisms in treating chronic myelogenous leukemia based on the 19 key gene nodes in the PPI and nine components of Qingdai. These studies have proved the reliability of network pharmacology. Based on these models, we proposed a new model with two main advantages. The first point was that our model considered whether the selected components are the better component group in the original formula. The second point was that we not only paid attention to the key gene nodes and component nodes in the complex network but also considered the coverage of the information of the original network after components screening. Our strategy can avoid information redundancy and noise as well as the loss of effective information.

In this study, we explored the potential coordinated functional space of PAR and ALD based on the C-T networks. We found that 341 targets were shared by the two herbs. It indicated the potential co-functional therapeutic effect of PAR and ALD in EMP. Then, we constructed the coordinated functional space prediction models and performed the knapsack algorithm to identify the key coordinated functional components of the potential coordinated functional space. Thirty-seven components were defined as the key coordinated functional components. The results of GO and KEGG pathway enrichment analysis on the targets of CFCG showed that the 426 targets were enriched in 86.96 and 97.92% of GO-BP terms and KEGG pathway of the overlap of RA-related genes and 924 targets of 126 active components. The CFCG identified by our method can cover the most function information of the original active components from EMP. The functional enrichment analysis results also indicated that the CFCG could coordinate the treatment of RA by regulating the genes that involved in biological processes and signaling pathways related to inflammation and immunity, such as oxygen levels (GO:0070482), response to hypoxia (GO:0001666), regulation of innate immune response (GO:0045088), immune response-activating cell surface receptor signaling pathway (GO:0002429), T cell activation (GO:0042110), PI3K-Akt signaling pathway (hsa04151), MAPK signaling pathway (hsa04010), TNF signaling pathway (hsa04668), and NF-κB signaling pathway (hsa04064). It has been reported that the PI3K-Akt signaling pathway is involved in regulating the expression of inflammatory cytokines and plays an important role in the development and progression of various types of inflammation (Liu et al., 2019; Gao et al., 2020). The development and progression of RA can be attenuated by inhibiting PI3K/Akt/mTOR signaling pathway activity (Wu et al., 2017; Liu et al., 2019; Li and Wang 2020). The MAPK signaling pathway can contribute to the development and progression of RA by mediating the proliferation and migration of RA fibroblast-like synoviocytes (Ralph and Morand 2008; Sujitha and Rasool 2017; Liu et al., 2018). The symptoms of RA, such as synovial tissue hyperplasia and articular cartilage tissue injury, can be relieved by inhibiting the MAPK signaling pathway (Bao et al., 2018; Liu et al., 2018; Yang et al., 2018). The TNF signaling pathway is associated with osteoarthritis activity and pathology, including bone loss, osteoblasts proliferation, and inflammation (Yu et al., 2018; Li et al., 2019; Jiang et al., 2020). The inhibition of the TNF signaling pathway can decrease inflammation and bone destruction (Yu et al., 2018). The NF-κB signaling pathway has been reported to be related to the pathogenesis of RA (Xia et al., 2018). It can regulate proliferation, apoptosis, and angiogenesis of human fibroblast-like synovial cells in RA (Zhang et al., 2017; Mitchell and Carmody 2018; Xia et al., 2018; Cao et al., 2019). Multiple studies have shown that T cell dysregulation appeared during the pathogenesis of RA (Fournier 2005; Larbi et al., 2008; Park et al., 2020). The activation and differentiation of T cells partly depend on the signal strength received by TCR (Goronzy and Weyand 2008), whereas altered TCR signaling thresholds can promote the occurrence and development of autoimmune arthritis (Larbi et al., 2008; Sakaguchi et al., 2012). RA is associated with uncontrolled transendothelial migration of leukocytes, and the cyclic adenosine 3',5'-monophosphate signaling pathway is involved in the molecular mechanisms that regulate the migration of leukocytes across the endothelium (Lorenowicz et al., 2006; Lorenowicz et al., 2007). These results demonstrated the reliability and accuracy of our strategy. Also, the result of molecular docking showed that the CFCG could bind well with the proteins coded by target genes in the comprehensive pathway and supported their important roles in the treatment of RA. All these results suggested the coordinated function mechanism of the CFCG identified from PAR and ALD in EMP treatment of RA. Finally, our experiment results also demonstrated the reliability and accuracy of the coordinated functional space prediction models and the knapsack algorithm.

In summary, we decoded and validated the molecular mechanisms of EMP in treating RA based on system pharmacological model, knapsack algorithm, molecular docking, and *in vitro* experiments. PAR and ALD play a coordinating role by regulating genes related to oxygen levels, immune response, synthesis of inflammatory mediators, and so on. Our strategy has the function of compound optimization and can further explore the coordinated mechanism of the CFCG, which was identified in the optimization process. Specifically, the components screened by the coordinated functional space prediction model can cover the most information of the original active components from EMP, and the reverse optimization method based on the targets of CFCG can also retain most targets of the screened components from our model. The identification of CFCG may contribute to improving our understanding of the coordinated mechanism of TCM in treating complex diseases. Moreover, the strategy can reduce the experiment time and cost of the studies on the mechanisms of TCM formulas. It can provide a reference for TCM mechanism research and ideas for the development of new drugs. However, our research lacks sufficient experimental verification. More components from CFCG and their targets should be selected to verify the reliability of our strategy. The determination of the content of herbs' components is also a difficult problem, and the effect of the components is related to the content. It requires further experimental verification. Our research mainly focuses on exploring the coordinated mechanism of herbs and the identification of CFCG. Although the CFCG plays a major therapeutic role, the effect of other components cannot be ignored. Further research is needed to analyze the effect of non-CFCG.

## Data Availability

The original contributions presented in the study are included in the article/Supplementary Material; further inquiries can be directed to the corresponding authors.
