# Corrigendum: An integrative pharmacology model for decoding the underlying Therapeutic Mechanisms of Ermiao Powder for Rheumatoid arthritis

**DOI:** 10.3389/fphar.2023.1302388

**Published:** 2023-10-09

**Authors:** Jie Wu, Kexin Wang, Qinwen Liu, Yi Li, Yingying Huang, Yujie Liu, Jieqi Cai, Chuanhui Yin, Xiaowei Li, Hailang Yu, Wei Meng, Handuo Wang, Aiping Lu, Yazi Li, Daogang Guan

**Affiliations:** ^1^ Department of Biochemistry and Molecular Biology, School of Basic Medical Science, Southern Medical University, Guangzhou, China; ^2^ Guangdong Province Key Laboratory of Single Cell Technology and Application, Guangzhou, China; ^3^ Neurosurgery Institute, Department of Neurosurgery, Zhujiang Hospital, Southern Medical University, Guangzhou, China; ^4^ Department of Radiology, Nanfang Hospital, Southern Medical University, Guangzhou, China; ^5^ Department of Obstetrics and Gynecology, Nanfang Hospital, Southern Medical University, Guangzhou, China; ^6^ Institute of Integrated Bioinformedicine and Translational Science, Hong Kong Baptist University, Hong Kong, China; ^7^ Guangdong-Hong Kong-Macau Joint Lab on Chinese Medicine and Immune Disease Research, Guangzhou, China

**Keywords:** rheumatoid arthritis (RA), traditional Chinese medicine (TCM), Ermiao Powder (EMP), coordinated functional components group (CFCG), coordinated molecular mechanisms

In the published article, there was an error in the **Author** list, and author “Qinwen Liu” was erroneously not listed as a co-first author. The corrected author list appears below.

“Jie Wu^†^, Kexin Wang^†^, Qinwen Liu^†^, Yi Li^†^, Yingying Huang, Yujie Liu, Jieqi Cai, Chuanhui Yin, Xiaowei Li, Hailang Yu, Wei Meng, Handuo Wang, Aiping Lu^*^, Yazi Li^*^, Daogang Guan^*^.”

The authors apologize for this error and state that this does not change the scientific conclusions of the article in any way. The original article has been updated.

