# Supplementary material for: An Integrative Pharmacology Model for Decoding the Underlying Therapeutic Mechanisms of Ermiao Powder for Rheumatoid Arthritis
Source: Front Pharmacol. 2022 Feb 23;13:801350. doi: 10.3389/fphar.2022.801350 (PMC8905663; doi:10.3389/fphar.2022.801350)
Supplement: Supplementary file 1 [file Table1.DOCX]

Supplementary Table 1. 187 Components in PAR and ALD.

| Herbs | Mol ID | Molcule name |
| --- | --- | --- |
| PAR | MOL001454 | berberine |
|  | MOL001458 | coptisine |
|  | MOL001965 | Dauricine (8CI) |
|  | MOL000200 | (S)-(+)-alpha-Phellandrene |
|  | MOL002329 | Javanicin |
|  | MOL000234 | L-Limonen |
|  | MOL002365 | (s)-carvone |
|  | MOL002635 | (±)-lyoniresinol |
|  | MOL002636 | Kihadalactone A |
|  | MOL002637 | Obacunoic acid |
|  | MOL013352 | Obacunone |
|  | MOL002639 | Obamegine |
|  | MOL002640 | phellavin |
|  | MOL002641 | Phellavin_qt |
|  | MOL002642 | phellodendrine |
|  | MOL002643 | delta 7-stigmastenol |
|  | MOL002644 | Phellopterin |
|  | MOL002645 | PEA |
|  | MOL002646 | Vanilloloside |
|  | MOL002647 | Vanillyl alcohol |
|  | MOL002648 | (4R)-limonene 1beta,2beta-epoxide |
|  | MOL002649 | Coniferin |
|  | MOL002650 | Coniferol |
|  | MOL002651 | Dehydrotanshinone II A |
|  | MOL002652 | delta7-Dehydrosophoramine |
|  | MOL002653 | Dictamine |
|  | MOL002654 | Amurensin |
|  | MOL002655 | Amurensin_qt |
|  | MOL002656 | dihydroniloticin |
|  | MOL002657 | hispidol B |
|  | MOL002658 | kihadalactone B |
|  | MOL002659 | kihadanin A |
|  | MOL002660 | niloticin |
|  | MOL002661 | nomilin |
|  | MOL002662 | rutaecarpine |
|  | MOL002663 | Skimmianin |
|  | MOL002664 | Fagarine |
|  | MOL002665 | Ferulic Acid |
|  | MOL002666 | Chelerythrine |
|  | MOL000449 | Stigmasterol |
|  | MOL002668 | Worenine |
|  | MOL002669 | Campesteryl ferulate |
|  | MOL002670 | Cavidine |
|  | MOL002671 | Candletoxin A |
|  | MOL002672 | Hericenone H |
|  | MOL002673 | Hispidone |
|  | MOL000347 | Syrigin |
|  | MOL000348 | 4-[(Z)-3-hydroxyprop-1-enyl]-2,6-dimethoxyphenol |
|  | MOL000358 | beta-sitosterol |
|  | MOL000622 | Magnograndiolide |
|  | MOL000741 | (2S,3S)-3,5,7-trihydroxy-2-(4-hydroxyphenyl)chroman-4-one |
|  | MOL000762 | Palmidin A |
|  | MOL000764 | magnoflorine |
|  | MOL000782 | Menisporphine |
|  | MOL000785 | palmatine |
|  | MOL000787 | Fumarine |
|  | MOL000790 | Isocorypalmine |
|  | MOL000908 | beta-elemene |
|  | MOL000069 | palmitic acid |
|  | MOL000098 | quercetin |
|  | MOL000115 | Undecenal |
|  | MOL000172 | Furol |
|  | MOL000197 | Myrcene |
|  | MOL000254 | eugenol |
|  | MOL000303 | caprylic acid |
|  | MOL000357 | Sitogluside |
|  | MOL000383 | D-Galacturonic acid, homopolymer |
|  | MOL000389 | FERULIC ACID (CIS) |
|  | MOL000508 | Friedelin |
|  | MOL000635 | vanillin |
|  | MOL000668 | PENTYLFURAN |
|  | MOL000675 | oleic acid |
|  | MOL000708 | WLN: VHR |
|  | MOL000716 | trans-2-nonenal |
|  | MOL000786 | STOCK1N-14407 |
|  | MOL000789 | jatrorrizine |
|  | MOL000794 | menisperine |
|  | MOL000860 | stearic acid |
|  | MOL000874 | paeonol |
|  | MOL000879 | methyl palmitate |
|  | MOL000924 | Mnk |
|  | MOL001131 | phellamurin_qt |
|  | MOL001218 | Pisol |
|  | MOL001377 | Oxophorone |
|  | MOL001396 | PENTADECYLIC ACID |
|  | MOL001455 | (S)-Canadine |
|  | MOL001457 | columbamine |
|  | MOL001752 | EUG |
|  | MOL001771 | poriferast-5-en-3beta-ol |
|  | MOL001867 | Isovanillin |
|  | MOL002176 | Methyl 3-furoate |
|  | MOL002331 | N-Methylflindersine |
|  | MOL002334 | Homocresol |
|  | MOL002522 | beta-Rhodinol |
|  | MOL002539 | Phlorol |
|  | MOL002891 | magnoflorine |
|  | MOL002894 | berberrubine |
|  | MOL002900 | Noroxyhydrastinine |
|  | MOL002901 | phellodendrine |
|  | MOL002902 | Ethyl caffeate |
|  | MOL002983 | Guasol |
|  | MOL002998 | IPH |
|  | MOL003050 | nonanoic acid |
|  | MOL003450 | dodec-2-enal |
|  | MOL003493 | naphthalene |
|  | MOL003959 | limonin |
|  | MOL003992 | 2-PENTADECANONE |
|  | MOL004119 | 5-METHYLFURFURAL |
|  | MOL004368 | Hyperin |
|  | MOL004474 | Maruzen M |
|  | MOL004479 | o-cresol |
|  | MOL004483 | CREOSOL |
|  | MOL004582 | Methyl naphthalene |
|  | MOL005438 | campesterol |
|  | MOL005928 | isoferulic acid |
|  | MOL006000 | Cyclopentenone |
|  | MOL006218 | Methyl caffeate |
|  | MOL006219 | Clorius |
|  | MOL006239 | Ptelein |
|  | MOL006276 | SMR000232320 |
|  | MOL006314 | Canthin-6-one |
|  | MOL006374 | 4,10-dimethylene-7-isopropyl-5(E)-cyclodecenol |
|  | MOL006384 | 4-[(1R,3aS,4R,6aS)-4-(4-hydroxy-3,5-dimethoxyphenyl)-1,3,3a,4,6,6a-hexahydrofuro[4,3-c]furan-1-yl]-2,6-dimethoxyphenol |
|  | MOL006392 | dihydroniloticin |
|  | MOL006394 | guanidine |
|  | MOL006401 | melianone |
|  | MOL006413 | phellochin |
|  | MOL006414 | 7-hydroxy-6-(2-hydroxyethyl)coumarin |
|  | MOL006422 | thalifendine |
|  | MOL006423 | vanilloloside |
|  | MOL006735 | Furfuranol |
|  | MOL007543 | cis-9-Hexadecenal |
|  | MOL008120 | (S)-4-Nonanolide |
|  | MOL008261 | 2,4,6-trimethyl-Octane |
|  | MOL008451 | Methyl atratate |
|  | MOL008653 | Acetylfuran |
|  | MOL010845 | candicine |
|  | MOL010927 | 2-undecenoic acid |
|  | MOL011089 | Homoveratrole |
|  | MOL013434 | Auraptene |
| ALD | MOL000114 | vanillic acid |
|  | MOL000162 | beta-Chamigrene |
|  | MOL000163 | Atractylodin |
|  | MOL000164 | atractylone |
|  | MOL000165 | 2-[(2S,5S,6S)-6,10-dimethylspiro[4.5]dec-9-en-2-yl]propan-2-ol |
|  | MOL000166 | ZINC01609418 |
|  | MOL000167 | 3β-hydroxyatractylone |
|  | MOL000168 | ()-2-Carene |
|  | MOL000169 | alpha-Guaiene |
|  | MOL000170 | guaiene |
|  | MOL000171 | Guaiol |
|  | MOL000172 | Furol |
|  | MOL000173 | wogonin |
|  | MOL000174 | (2E,8E)-9-(2-furyl)nona-2,8-dien-4,6-diyn-1-ol |
|  | MOL000175 | cyperene |
|  | MOL013068 | Oroxindin |
|  | MOL000043 | atractylenolide i |
|  | MOL000178 | atractylenolide iii |
|  | MOL000179 | 2-Hydroxyisoxypropyl-3-hydroxy-7-isopentene-2,3-dihydrobenzofuran-5-carboxylic |
|  | MOL000180 | Aractylenolide II |
|  | MOL000181 | atractylenolide III |
|  | MOL000182 | Atractyloyne |
|  | MOL000183 | Beta- Eudesmol |
|  | MOL000184 | NSC63551 |
|  | MOL000185 | Stigmasterol 3-O-beta-D-glucopyranoside |
|  | MOL000186 | Stigmasterol 3-O-beta-D-glucopyranoside_qt |
|  | MOL000187 | butenolide B |
|  | MOL000188 | 3β-acetoxyatractylone |
|  | MOL000189 | acetyl atractylodinol |
|  | MOL000190 | 3,5-dimethoxy-4-glucosyloxyphenylallylalcohol |
|  | MOL000191 | 3,5-dimethoxy-4-glucosyloxyphenylallylalcohol_qt |
|  | MOL000192 | 2-(1,4a-dimethyl-2,3-dihydroxydecahydronaphthalen-7-yl)isopropyl glucoside |
|  | MOL000193 | (Z)-caryophyllene |
|  | MOL000194 | patchoulene |
|  | MOL000024 | alpha-humulene |
|  | MOL000032 | beta-Eudesmol |
|  | MOL000034 | 2-[(1R,3S,4S)-3-isopropenyl-4-methyl-4-vinylcyclohexyl]propan-2-ol |
|  | MOL000044 | Atractylenolide II |
|  | MOL000060 | selina-4(14),7(11)-dien-8-one |
|  | MOL000084 | beta-daucosterol |
|  | MOL000085 | beta-daucosterol_qt |
|  | MOL000086 | (24S)-5beta-Stigmastan-3beta-ol |
|  | MOL000087 | beta-sitosterol 3-O-glucoside |
|  | MOL000088 | beta-sitosterol 3-O-glucoside_qt |
|  | MOL000091 | daucosterin |
|  | MOL000092 | daucosterin_qt |
|  | MOL000093 | daucosterol |
|  | MOL000094 | daucosterol_qt |
|  | MOL000095 | delta 7-stigmastenol |
|  |  |  |
| Common in the 2 herbs | MOL000172 | Furol |
|  | MOL000095/ MOL002643 | delta 7-stigmastenol |
